# How different combinations of comorbidities affect healthcare use by elderly patients with obstructive lung disease

**DOI:** 10.1038/s41533-021-00242-y

**Published:** 2021-05-25

**Authors:** Alessandra Buja, Andrea Bardin, Giulia Grotto, Stefania Elvini, Pietro Gallina, Giulia Zumerle, Patrizia Benini, Domenico Scibetta, Vincenzo Baldo

**Affiliations:** 1 Department of Cardiologic, Vascular and Thoracic Sciences, and Public Health, University of Padua, Padova, Italy; 2 School of Specialization in Hygiene, Preventive Medicine and Public Health, University of Padua, Padova, Italy; 3 Regione Veneto, AULSS 6 Euganea, Padova, Italy

**Keywords:** Health services, Public health

## Abstract

Previous research had shown the number of comorbidities is a major factor influencing the burden of care for elderly patients with obstructive lung disease (OLD). This retrospective cohort study on a large population of elderly patients (age > 65 years) with OLD in northern Italy measures the use of healthcare resources associated with the most frequent combinations of comorbidities and investigates the most common reasons for hospitalization. Total health costs, pharmacy costs, emergency department (ED) visits, outpatient visits, and hospital admissions are assessed for every subject. The most common causes of hospitalization by a number of comorbidities and the most common sets of three comorbidities are identified. For each comorbidity group, we rank a list of the most frequent causes of hospitalization, both overall and avoidable with effective ambulatory care. A small group of patients suffering from major comorbidities accounts for the use of most healthcare resources. The most frequent causes of hospitalization are respiratory failure, heart failure, chronic bronchitis, and bronchopneumonia. The most common conditions manageable with ambulatory care among causes of hospitalizations are heart failure, bacterial pneumonia, and COPD. The set of three comorbidities responsible for the highest average total costs, and the highest average number of hospitalizations and outpatient visits comprised hypertension, cardiac arrhythmias, and heart failure. The main reasons for hospitalization proved to remain linked to heart failure and acute respiratory disease, regardless of specific combinations of comorbidities. Based on these findings, specific public health interventions among patients with OLD cannot be advised on the basis of specific sets of comorbidities only.

## Introduction

Among chronic conditions, asthma and chronic obstructive pulmonary disease (COPD) are by far the most common obstructive lung diseases (OLD). Both are characterized by inspiratory and expiratory airflow limitation^1^. Asthma and COPD pose significant public health problems everywhere, and particularly in high-income countries^2^. In terms of costs, COPD is the obstructive pulmonary syndrome with the greatest impact due to the disease’s clinical characteristics and the average age of the patients affected. COPD nonetheless goes largely underdiagnosed, especially in the early clinical stages^3,4^. The prevalence of COPD varies from one country to another, and by age and sex. A multicenter study in Italy found a prevalence of COPD ranging from 3.3% in young adults (20–44 years) to 13.3% among the elderly (65–84 years). On the other hand, the prevalence of asthma alone was found to range from 8.2% in young adults (20–44 years old) to 2.9% among the elderly (65–84 years). On top of that, a particular form of asthma combined with COPD (asthma–COPD overlap syndrome, ACOS) affects an additional 1.6% of young adults and 4.5% of older adults^5^.

Several chronic diseases are commonly associated with COPD. It is estimated that at least 80% of patients have at least one additional chronic condition, and there is an association between the severity of their COPD and the burden of their comorbidities^6^. Multimorbidity (i.e., the simultaneous presence of two or more chronic conditions in the same individual) is known to have a heavy impact on survival, quality of life, and use of healthcare services. The literature on multimorbidity has highlighted its association with a greater risk of premature death, hospitalization, disability, poor functional status, low quality of life, and adverse drug reactions. These associations become stronger the larger the number of concomitant chronic diseases^7–9^. Multimorbidity demands a great commitment of diagnostic, outpatient, and hospital services, and polypharmacy, and the usage of care services gradually increases with the number of chronic diseases^10^. According to some studies, the relationship between the number of comorbidities and the related public health costs is exponential, with a tendency for the costs to double with each additional disease in a given individual^11^. In particular, previous studies on patients with COPD found that comorbidities make an important contribution to the total costs^10,12^. Often amounting to more than the costs due to the respiratory condition itself^13,14^. In line with these reports, comorbidities have also emerged as one of the main drivers of intense healthcare use by patients with COPD^15^. Similar findings have been published regarding patients with ACOS^16^ and asthma^17^. As concerns, specific comorbidities, an American study on COPD patients found a particularly strong link between their health service usage and hypertension, diabetes mellitus, coronary artery disease, and heart failure^18^. Hospital care is a major contributor to healthcare costs, especially for multimorbid patients. Multimorbidity is associated with more hospital stays, and the number of admissions increases with the number of chronic conditions^19,20^. Few studies have looked for an association between specific comorbidities and the risk of hospitalization for patients with OLD. One American study identified heart failure, coronary, and cerebrovascular diseases as strongly associated with all-cause hospitalizations among such patients^21^. A Spanish study likewise found that cardiovascular comorbidities contributed the most to overall hospitalization risk among patients with ACOS^16^.

In the light of these considerations, and with a view to optimizing healthcare spending, there is a clear need to adopt the most appropriate strategies for managing the growing burden of multimorbidity, and containing the use of hospital care in particular. To this end, the role of primary care in the management of chronic conditions seems pivotal. An indicator frequently used when assessing a population’s interaction with health services and the effectiveness of a primary care system is “avoidable hospitalization”, defined as the proportion of hospital admissions that could be avoided by optimal out-of-hospital care^22^. While avoidable hospitalizations can be used as an indicator of the effectiveness of primary care, it is also strongly related to multimorbidity. One previous study found that the risk of avoidable hospital admissions increases by a factor of 1.35 for each additional chronic condition in a given patient^23^.

As described above, only a few studies have been conducted on the relationship between specific comorbidities and the use of healthcare resources by patients with OLD. To the best of our knowledge, little research has been done on the most common reasons for such patients’ hospitalization, and less still with a specific focus on hospital admissions that could be avoided with appropriate primary care.

The present study analyses a large cohort of elderly subjects in Northeast Italy, focusing on possible associations between different comorbidities and healthcare use among patients with OLD. It aims to measure the use of healthcare resources, and the most frequent causes of hospitalization, in relation to different combinations of comorbidities, pinpointing potentially avoidable hospital admissions to develop more adequate public health strategies for the prevention and the management of these events in a primary care setting.

## Results

### Characteristics of the sample

Of the 120,181 individuals with OLD assisted by the LHU, 37,915 were at least 65 years old and formed the study sample on which subsequent analyses were conducted. Since the population served by the LHU amounted to 936,740 in 2017, our sample amounted to 4.05% of the reference population. The average age of our sample was 77.33 years, and women made up 54.5% of the total.

This sample included 35,234 patients with at least one comorbidity (92.9% of the total). In other words, only a small proportion of them had OLD alone. The other patients had an average number of three comorbidities, with a very slight sex-related difference (2.95 for males, 3.04 for females). Patients with five or more comorbidities, making up almost 21% of the sample (10.4% had five comorbidities, and 10.5% had six or more), accounted for almost 40% of total costs and almost 50% of hospital admissions.

### Costs and health service usage by the most common comorbidity triplets

Among the patients with three chronic diseases in addition to OLD, the six most common combinations of diseases in these comorbidity triplets were identified and analyzed by health resource usage, healthcare costs, and mortality (Table 1). Hypertension was understandably involved in all six combinations identified, given its higher prevalence than any of the other conditions considered in our study sample. The group of OLD patients who also had hypertension, cardiac arrhythmias, and heart failure coincided with the highest average total costs per patient (€3733), and the highest average number of hospitalizations (0.47) and outpatient visits (11.2). The triplet of comorbidities with the highest costs for drugs and emergency department (ED) services included hypertension, diabetes, and dyslipidemia, with an average cost per patient of €1277, and an average of 0.79 ED visits per year.

**Table 1:** Use of healthcare services and costs by the most common comorbidity triplets in patients with OLD over 65 years old.

Comorbidity triplets		Hypertension + osteoporosis + dyslipidemia	Hypertension + diabetes + dyslipidemia	Hypertension + ischemic heart disease + dyslipidemia	Hypertension + heart failure + arrhythmias	Hypertension + osteoporosis + depression	Hypertension + heart failure + depression	*P*-value
No. of patients		918	648	480	449	381	289	
Sex	F % (*n*)	86.49 (794)	35.03 (227)	16.88 (81)	42.98 (193)	89.24 (340)	80.97 (234)	<0.001*
	M % (*n*)	13.51 (124)	64.97 (421)	83.13 (399)	57.02 (256)	10.76 (41)	19.03 (55)	
Age	Mean	74.92	74.78	74.39	82.38	76.69	81.61	<0.001**
	Median	74	74	74	83	76	82	
	(IQR)	(70–79)	(70–79)	(69–79)	(78–88)	(70–82)	(74–88)	
Total cost (€)	Mean	1833.42	3224.87	2624.17	3732.95	2231.16	3202.67	<0.001**
	Median	1041.77	783.69	1456.70	1824.13	1150.28	1389.51	
	(IQR)	(646–1798)	(1298–2081)	(850–2601)	(896–4277)	(674–2048)	(826–3493)	
Pharmacy costs (€)	Mean	704.14	1277.18	965.06	817.59	687.96	1062.35	<0.001**
	Median	527.285	441.015	815.11	585.5	516.15	660.35	
	(IQR)	(334–853)	(817–1258)	(494–1218)	(329–1168)	(325–832)	(405–1111)	
Emergency (ED) visits	Mean	0.32	0.79	0.39	0.68	0.45	0.41	<0.001**
	Median	0	0	0	0	0	0	
	(IQR)	(0–0)	(0–0)	(0–1)	(0–1)	(0–1)	(0–1)	
Outpatient visits	Mean	7.85	5.94	7.59	11.21	8.66	8.43	<0.001**
	Median	7	4	6	10	7	6	
	(IQR)	(3–10)	(6–10)	(3–10)	(4–16)	(3–13)	(3–12)	
Hospital admissions	Mean	0.11	0.40	0.19	0.47	0.13	0.28	<0.001**
	Median	0	0	0	0	0	0	
	(IQR)	(0–0)	(0–0)	(0–0)	(0–1)	(0–0)	(0–0)	
Crude mortality rate (%)		0.11	1.40	0.84	9.88	2.39	6.86%	<0.001***

*Chi square, **Kruskal-Wallis, ***Fisher’s test.

### Hospitalizations

In the 2-year period considered (2017–2018) there were 28,695 hospital admissions for patients in the study sample, and 5293 of them were avoidable (18.4% of the total). The most frequent reasons for their hospitalization, based on primary diagnosis at discharge, were: “other lung diseases” (2382 hospitalizations, 8.3% of the total); heart failure (2295 hospitalizations, 8%); chronic bronchitis (803 hospitalizations, 2.8%); and bronchopneumonia (766 hospitalizations, 2.7%). The most common ACSCs involved were: heart failure (2328 hospitalizations, 44% of all avoidable admissions); bacterial pneumonia (1302 hospitalizations, 24.6% of avoidable admissions); and COPD (795 hospitalizations, 15% of avoidable admissions). Taken together, these three conditions accounted for 83.6% of avoidable hospitalizations in our sample.

Table 2 shows the most common reasons for overall (total) and avoidable hospital admissions. relating to the primary diagnosis on the hospital discharge records across our study sample. The overall count is based on the first 3 digits of the ICD-9 code.

**Table 2:** Most frequent reasons for hospitalization (total and avoidable) by primary diagnosis in the whole study sample, years 2017–2018.

Total hospitalizations	*N*	%	Avoidable hospitalizations (for ACSCs)	*N*	%
518 Other lung diseases	2382	8.3	Heart failure*	2328	44.0
428 Heart failure	2295	8.0	Bacterial pneumonia*	1302	24.6
491 Chronic bronchitis	803	2.8	COPD**	795	15.0
485 Bronchopneumonia, unspecified agent	766	2.7	Urinary tract infections**	203	3.8
427 Cardiac arrhythmias	624	2.2	Acute bronchitis**	148	2.8
715 Generalized arthrosis	582	2.0	Angina*	121	2.3
V43 Substituted organ or tissue	541	1.9	Dehydration/hypovolemia**	117	2.2
410 Acute myocardial infarction	535	1.9	Diabetes mellitus**	78	1.5
162 Malignant tumors of the larynx	525	1.8	Epilepsy*	75	1.4
820 Fracture of the femoral neck	476	1.7	Asthma*	45	0.9

*Roos et al. (adaptations of Billings’ definitions), **Fransoo et al.

The hospitalizations with a diagnosis of “other lung diseases” were further broken down to investigate the specific conditions behind them (complete results of the analysis not shown). Of the 2382 cases with this diagnostic code, 2308 (96.9%) were labeled as either acute or chronic respiratory failure.

The analysis of the most frequent reasons for hospitalization for specific combinations of chronic diseases among OLD patients with three comorbidities (*triplets*) pointed, here again, to heart failure and acute respiratory disease being among the main drivers of hospitalization in all triplets (Table 3).

**Table 3:** Most frequent reasons for total and avoidable hospital admissions for patients with OLD by comorbidity triplets (sets of three different comorbidities) in 2017–2018, showing absolute numbers and percentages of total.

Most frequent diagnoses (3-digit ICD-9)	*N*	%	Most frequent ACSCs	*N*	%
*Comorbidity triplet: hypertension, heart failure, arrhythmias*					
428 Heart failure	71	22.6	Heart failure	81	61.4
518 Other lung diseases	55	17.5	Bacterial pneumonia	30	22.7
485 Bronchopneumonia, unspecified agent	16	5.1	COPD	16	12.1
491 Chronic bronchitis	16	5.1	Asthma	2	1.5
402 Hypertensive heart disease	10	3.2	Urinary tract infections	2	1.5
*Comorbidity triplet: hypertension, diabetes, dyslipidemia*					
428 Heart failure	6	4.9	Heart failure	7	29.2
491 Chronic bronchitis	6	4.9	COPD	6	25.0
715 Arthrosis	5	4.1	Bacterial pneumonia	4	16.7
518 Other lung diseases	4	3.3	Urinary tract infections	3	12.5
786 Symptoms relating to the respiratory system (…)	4	3.3	Diabetes mellitus	2	8.3
*Comorbidity triplet: hypertension, osteoporosis, dyslipidemia*					
518 Other lung diseases	12	11.0	Bacterial pneumonia	7	46.7
786 Symptoms relating to the respiratory system (…)	6	5.5	COPD	4	26.7
715 Osteoarthritis	5	4.6	Angina	2	13.3
427 Cardiac arrhythmias	4	3.7	Cellulite	1	6.7
560 Bowel obstruction with no hernia	4	3.7	Heart failure	1	6.7
*Comorbidity triplet: hypertension, ischemic heart disease, dyslipidemia*					
410 Acute myocardial infarction	12	10.7	Angina	5	38.5
411 Other acute and subacute forms of ischemic heart disease	8	7.1	Heart failure	3	23.1
414 Other forms of chronic ischemic heart disease	7	6.3	Bacterial pneumonia	2	15.4
518 Other lung diseases	5	4.5	Cellulite	1	7.7
413 Angina pectoris	4	3.6	COPD	1	7.7
*Comorbidity triplet: hypertension, heart failure, osteoporosis*					
428 Heart failure	21	14.5	Heart failure	22	46.8
518 Other lung diseases	17	11.7	COPD	13	27.7
491 Chronic bronchitis	13	9.0	Bacterial pneumonia	4	8.5
434 Occlusion of cerebral arteries	5	3.4	Cellulite	2	4.3
276 Disorders of fluids, electrolytes and acid-base balance	4	2.8	Dehydration/hypovolemia	2	4.3
*Comorbidity triplet: hypertension, depression, osteoporosis*					
518 Other lung diseases	11	11.1	Bacterial pneumonia	4	36.4
296 Episodic mood disorders	9	9.1	Acute bronchitis	2	18.2
820 Fracture of the femoral neck	7	7.1	Asthma	1	9.1
276 Disorders of fluids, electrolytes and acid-base balance	4	4.0	COPD	1	9.1
300 Anxiety, dissociative and somatoform disorders	4	4.0	Urinary tract infections	1	9.1

## Discussion

The present study evidenced that a small group of patients suffering from major comorbidities accounted for most of the healthcare resource consumption. The most frequent reasons for hospitalization were “other lung diseases” (mainly acute and chronic respiratory failure), heart failure, chronic bronchitis, and bronchopneumonia. The most common conditions manageable with ambulatory care among the avoidable hospitalizations were heart failure, bacterial pneumonia, and COPD.

Our results show that, among multimorbid patients with OLD, a combination of hypertension, cardiac arrhythmias, and heart failure was associated with the highest average total costs, and the largest average number of hospitalizations and outpatient visits, among patients with three comorbidities at least. These results suggest that cardiovascular comorbidities may be the main drivers of healthcare usage and costs among OLD patients. This is in line with the findings of a large American study on COPD patients, which showed a link between hypertension, diabetes mellitus, coronary artery disease and heart failure and intense health service usage^18^. Our analysis on hospital admissions showed that the most common causes for admission were acute or chronic respiratory failure, followed by heart failure, chronic bronchitis and bronchopneumonia. Similarly, the ACSCs most frequently seen in this sample were heart failure, bacterial pneumonia and COPD. These same conditions were often the reason for hospital admissions (in total and for ACSCs) for OLD patients with multiple comorbidities. The literature on the influence played by specific comorbidities on the risk of hospitalization among patients with OLD is limited. One large American study which looked into the association between comorbidities and all-cause hospitalizations have found heart failure, and coronary and cerebrovascular diseases to be the strongest associated comorbidity^21^. Cardiovascular comorbidities were found to contribute the most to overall hospitalization risk among patients with ACOS in at least one recent study^16^. Several associated conditions were also investigated as risk factors for asthmatic exacerbations leading to hospitalization^24^. but the population considered was not comparable with that of the present study. With regard to the most frequent causes of hospitalization, previous studies found dyspnea and respiratory infections to be the most common issues among patients with COPD^25^. That said, the lack of significant literature on the associations between specific comorbidities and hospitalization rates among patients with asthma makes it hard to say how grouping together subjects with COPD and asthma might have affected our results. It is possible, because of some risk factors associated with COPD such as smoking, that the subgroup of patients with COPD is at higher risk for hospitalization compared to the subgroup with asthma.

Identifying subgroups of patients with OLD who are at higher risk of making intensive use of healthcare services, and clarifying the main reasons why patients with OLD are hospitalized (as a whole and for ACSCs) may be useful steps towards the design of better policies. In fact, it could help decision-makers to develop adequate public health strategies for the prevention and the management of this type of chronic disease and the comorbidities often associated with it. Since hospital care is a major contributor to the rising total costs of healthcare services^26^. it would be useful to focus on preventing unnecessary hospital admissions targeting high-risk OLD patients whose comorbidities make them more likely to be hospitalized. Examples of public health interventions that have been proved efficacious in the reduction of hospitalization among multimorbid patients are self-management programs for patients with COPD^27^ and case management for patients with heart failure^28,29^.

The present study has some strengths and some methodological limitations as well, that need to be discussed. Analyzing our whole reference population, and using independently collected data from current administrative and public health sources minimized the risk of selection bias. The use of real-world data also generated an undistorted picture of actual healthcare service usage and costs for people with OLD. On the other hand, by pooling together patients with different forms of OLD (asthma and COPD), we may have failed to recognize specific patterns of comorbidities and health service usage due to differences in the pathogenesis of these diseases. Relying on pharmaceutical prescriptions to associate patients with their comorbidities can also lead to errors (missing or inappropriate diagnoses): whatever pharmaceutical prescription threshold (or other criterion) we may associate with a given disease may prove too high for some patients, and too low for others. That said, among the comorbidities that we considered here, only dyslipidemia and thyroid dysfunction were identified on the basis of pharmaceutical prescriptions alone. For all the other conditions, reference was also made to the EDC code. Using administrative data could also have led to some medical conditions being under-represented, but any selection of diseases would inevitably be arbitrary to some degree (and the comparison we drew with the literature on multimorbidity seems encouraging).

Considering a 2-year period (2017–2018) for the analysis of hospital admissions, whereas diagnoses of chronic conditions are updated to 2017, may be a further limitation. On the one hand, it increases the number of events, making our results more robust. This is especially relevant when breaking down the population in smaller subgroups, which can easily result in a very small number of hospitalizations for specific causes. On the other hand, this may have led to a bias in those cases where a new chronic condition has appeared during the second year of the 2-year period considered (2018). However, we are confident this is not a very large bias: due to the very nature of chronic conditions, it is more likely that a hospitalization event in 2018 is tied to chronic conditions accumulated through one’s lifetime and present in 2017, than tied to a chronic condition diagnosed for the first time in 2018.

A further important aspect concerns the absence of socio-economic variables associated with our patients (as they are not recorded in the ACG database). Failure to adjust for these variables may have introduced confounding biases.

When hospital admissions were analyzed in detail, our study found that heart failure, COPD exacerbation, and pneumonia were by far the most common reasons why patients with OLD were hospitalized, regardless of how many comorbidities they might have had, and in what combinations. These results reinforce the need for health services to manage this patient population better, especially as they grow older and become increasingly burdened with multiple medical conditions. Further research is needed to back up our findings, and to identify specific subgroups of OLD patients, as doing so could point to the most effective public health interventions for managing such chronic diseases, and for measuring their impact in terms of cost-effectiveness.

## Methods

### Context

The Italian National Health System (NHS) is a mainly public system financed by general taxation. All residents registered with the country’s NHS (be they Italians or regular immigrants) can access all healthcare services free of charge or by paying a small fee. Regional authorities plan and organize healthcare facilities and activities through their regional health departments in accordance with a national health plan designed to assure an equitable provision of comprehensive care throughout the country. They coordinate and control local health units (LHUs) that plan and deliver health services, primary care, and hospital care to their local communities, based on a regional health plan. LHUs may provide these services directly with their own facilities and personnel, or outsource them to other service providers. All residents registered with the country’s NHS are assigned a general practitioner (GP) of their choice. GPs are self-employed, independent physicians. They also serve as NHS gatekeepers, with a pivotal role in managing care for chronic diseases, such as OLD, as they establish care programs and treatment pathways for individual patients.

### Study design

This is a retrospective cohort study conducted using anonymized administrative data regarding patients of an LHU, the *Azienda ULSS 6 Euganea* in the Veneto Region of North-East Italy, which—as at the end of 2017—served a population of about 936,740 individuals.

### Data sources

Two digital archives of the *Azienda ULSS 6 Euganea* were used for this study: one based on the Adjusted Clinical Group (ACG) system (updated to 2017), and the other on hospital discharge records.

The database of hospital records for patients discharged in 2017 and 2018 was considered in our study. It contains the following information for all hospital admissions involving people served by the LHU: date of admission and discharge; primary and secondary diagnoses recorded at time of discharge.

The ACG is a population risk stratification method used internationally to characterize multimorbidity on the strength of routinely collected administrative data, which are pooled using record linkage^30^. It has been used in the Veneto Region since 2012^31^ and collects information at the individual patient level, including hospital stays, diagnoses allowing for fiscal exemptions, outpatient visits, ED visits, drug prescriptions, home care visits, mental health, rare diseases, outpatient rehabilitation. A detailed list of the information retrieved from the ACG database and used for the purposes of this study is provided in Supplementary Table 1.

A variable number of chronic conditions could be associated with each patient, based on the EDCs and Rx-MGs drawn from the ACG database. For the purposes of our subsequent analyses, 15 comorbidities were considered: heart failure, hypertension, ischemic heart disease, cardiac arrhythmias, cerebrovascular disease, diabetes mellitus, osteoporosis, cancer, Parkinson’s disease, dementia and delirium, depression, chronic kidney failure, rheumatoid arthritis, dyslipidemia, and thyroid dysfunction. Our choice of comorbidities to consider was guided by two main criteria based on existing epidemiological knowledge: the prevalence in the elderly, and the burden on the overall health of this segment of the population.

We then identified the most commonly occurring combinations of three comorbidities (triplets) in patients who had three chronic diseases in addition to OLD.

### Inclusion criteria

All the data used were anonymous and linked to a unique identification code for each individual. Only patients meeting the following inclusion criteria were selected from the initial ACG database: (1) residents in the area served by the *Azienda ULSS 6 Euganea*; (2) age over 64 years in 2017; and (3) a diagnosis of COPD or asthma according to data collected and processed by the ACG system. Patients with either of these diagnoses were included because they often overlapped or were difficult to tell apart using our administrative (not clinical) database. This is largely because the drugs used to treat the two conditions belong to much the same pharmacological classes, and this can lead to one being misdiagnosed as the other. A differential diagnosis between the two conditions is difficult even at clinical level^32^, and older people may have both^5^. No exclusion criteria were adopted for the present study.

### Outcomes

The healthcare consumption variables derived from the ACG database for the year 2017 were: total healthcare costs; costs of pharmaceuticals; and numbers of ED visits, outpatient visits and hospital admissions. The database of hospital discharge records for the 2-year period (2017–2018) was linked to the ACG database via the users’ unique identification codes, and provided information about hospital admissions stratified by: (1) number of comorbidities and (2) for patients with three comorbidities in addition to OLD, the six most common combinations of their comorbidity triplets.

Analyses were run separately for total hospitalizations and for ACSCs, as proposed by Billings for adult patients^22^. For the ACSCs we used the 3-digit adaptations applied by Roos et al.^33^ to the ICD-9 codes describing reasons for hospitalization because the prevalence of these conditions based on the first 3 digits of the ICD-9 codes differs little from the figure obtained with Billings’ original definitions. The only exception concerns heart failure, for which the original definition was adopted. Since we felt that Billings’ definitions would overlook some important causes of hospitalization in our population, we opted to consider six additional ACSCs drawn from the list proposed by Fransoo et al.^34^ were also included, chosen for their relevance to the adult population. The ACSCs thus considered in our analysis of avoidable hospitalizations were: asthma, angina, pelvic inflammatory disease, gastroenteritis, heart failure, upper airway infections, epilepsy, bacterial pneumonia, tuberculosis, dental conditions, and cellulite (as proposed by Billings et al.^22^); plus, COPD, acute bronchitis, diabetes mellitus, hypoglycemia, urinary tract infection, and dehydration/hypovolemia (drawn from Fransoo et al.^34^).

### Statistical methods

First, a descriptive analysis of the population was carried out, examining their demographic characteristics (sex, age), and a number of comorbidities. A descriptive analysis was conducted for absolute and relative frequencies of the categorical variables, calculating the means and standard deviations for quantitative variables (age, number of comorbidities). The medians and interquartile ranges (IQRs) were also calculated for variables with a non-normal distribution. The population was divided into categories based on a number of comorbidities (i.e., chronic diseases in addition to OLD). The subpopulation of patients with triplets of comorbidities in addition to OLD was divided according to the comorbidities involved. A bivariate analysis was also conducted to highlight differences in healthcare resource consumption and costs between these comorbidity triplet groups. For each group we ranked and listed the 10 most frequent reasons for hospitalizations (primary diagnoses), distinguishing between total and avoidable hospitalizations.

### Ethical issues

The data analysis was performed on anonymized aggregate data with no chance of individuals being identifiable. The study complied with the Declaration of Helsinki, and with resolution No. 9/2016 of the Italian Guarantor for the Protection of Personal Data, which also confirmed the allowability of processing personal data for medical, biomedical, and epidemiological research, and that data concerning people’s health status can be used in aggregate form in scientific studies.

Permission to use unidentifiable individual data extracted from administrative databases was granted by the *Azienda ULSS 6—Euganea*. To ensure confidentiality and anonymity, the Veneto Regional Authority removes all direct identifiers (e.g., NHS code numbers) and replaces them with a code number in all datasets to retain the opportunity to link data from different administrative databases.

### Reporting summary

Further information on research design is available in the Nature Research Reporting Summary linked to this article.

## Supplementary information

Reporting Summary

Supplementary Information

## Data Availability

The data that support the findings of this study are available from AULSS 6 Euganea, Regione Veneto, but restrictions apply to the availability of these data, which were used under license for the current study, and so are not publicly available. Data are however available from the corresponding author (alessandra.buja@unidp.it) upon reasonable request and with permission of AULSS 6 Euganea, Regione Veneto.

## References

[1] Osadnik CR, Singh S (2019). Pulmonary rehabilitation for obstructive lung disease. Respirology.

[2] GBD Chronic Respiratory Disease Collaborators. (2020). Prevalence and attributable health burden of chronic respiratory diseases, 1990–2017: a systematic analysis for the Global Burden of Disease Study 2017. Lancet Respir. Med..

[3] Rennard S, Decramer M, Calverley PM, Pride NB, Soriano JB, Vermeire PA, Vestbo J (2002). Impact of COPD in North America and Europe in 2000: subjects’ perspective of Confronting COPD International Survey. Eur. Respir. J..

[4] Dal Negro RW, Rossi A, Cerveri I (2003). The burden of COPD in Italy: results from the Confronting COPD survey. Respir. Med..

[5] De Marco R, Pesce G, Marcon A (2013). The coexistence of asthma and chronic obstructive pulmonary disease (COPD): prevalence and risk factors in young, middle-aged and elderly people from the general population. PLoS ONE.

[6] Putcha N, Drummond MB, Wise RA, Hansel NN (2015). Comorbidities and chronic obstructive pulmonary disease: prevalence, influence on outcomes, and management. Semin. Respir. Crit. Care Med..

[7] Salive ME (2013). Multimorbidity in older adults. Epidemiol. Rev..

[8] Violan C (2014). Prevalence, determinants and patterns of multimorbidity in primary care: a systematic review of observational studies. PLoS ONE.

[9] European Chronic Diseases Alliance. Joint Statement on “Improving the employment of people with chronic diseases in Europe”. 2017. https://ec.europa.eu/health/sites/health/files/policies/docs/2017_chronic_framingdoc_en.pdf (Accessed May 2021).

[10] Buja (2020). Healthcare service usage and costs for elderly patients with obstructive lung disease. Int. J. Chronic Obstr. Pulm. Dis..

[11] Lehnert T (2011). Review: health care utilization and costs of elderly persons with multiple chronic conditions. Med. Care Res. Rev..

[12] Lisspers K, Larsson K, Johansson G (2018). Economic burden of COPD in a Swedish cohort: the ARCTIC study. Int. J. Chron. Obstruct. Pulmon. Dis..

[13] Jansson SA, Backman H, Rönmark E, Lundbäck B, Lindberg A (2015). Hospitalization due to co-morbid conditions is the main cost driver among subjects with COPD—a report from the population-based OLIN COPD Study. COPD.

[14] Lin PJ, Shaya FT, Scharf SM (2010). Economic implications of comorbid conditions among Medicaid beneficiaries with COPD. Respir. Med..

[15] Simon-Tuval T, Scharf SM, Maimon N, Bernhard-Scharf BJ, Reuveni H, Tarasiuk A (2011). Determinants of elevated healthcare utilization in patients with COPD. Respir. Res..

[16] van Boven JF, Román-Rodríguez M, Palmer JF, Toledo-Pons N, Cosío BG, Soriano JB (2016). Comorbidome, pattern, and impact of asthma-COPD overlap syndrome in real life. Chest.

[17] Ban G-Y, Trinh THK, Ye Y-M, Park H-S (2016). Predictors of asthma control in elderly patients. Curr. Opin. Allergy Clin. Immunol..

[18] Westney G, Foreman MG, Xu J, Henriques King M, Flenaugh E, Rust G (2017). Impact of comorbidities among Medicaid enrollees with chronic obstructive pulmonary disease, United States, 2009. Prev. Chronic Dis..

[19] Condelius A, Edberg AK, Jakobsson U, Hallberg IR (2008). Hospital admissions among people 65+ related to multimorbidity, municipal and outpatient care. Arch. Gerontol. Geriatr..

[20] Nagl A, Witte J, Hodek JM, Greiner W (2012). Relationship between multimorbidity and direct healthcare costs in an advanced elderly population. Results of the PRISCUS trial. Z. Gerontol. Geriatr..

[21] Schwab P, Dhamane AD, Hopson SD (2017). Impact of comorbid conditions in COPD patients on health care resource utilization and costs in a predominantly Medicare population. Int. J. Chron. Obstruct. Pulmon. Dis..

[22] Billings J, Zeitel L, Lukomnik J, Carey TS, Blank AE, Newman L (1993). Impact of socio-economic status on hospital use in New York City. Health Aff..

[23] Dantas I, Santana R, Sarmento J, Aguiar P (2016). The impact of multiple chronic diseases on hospitalizations for ambulatory care sensitive conditions. BMC Health Serv. Res..

[24] Porsbjerg C, Menzies-Gow A (2017). Co-morbidities in severe asthma: clinical impact and management. Respirology.

[25] Ramdani, I., Pescatore K. A. & Bouazza, B. Causes of hospitalization and characteristics of Algerian chronic obstructive pulmonary disease patients in Tizi-Ouzou: a retrospective study. *Monaldi Arch. Chest Dis.* **90**, 10.4081/monaldi.2020.1317 (2020). 10.4081/monaldi.2020.1317 32657106

[26] *Istituto Nazionale di Statistica (ISTAT). Il sistema dei conti della sanità per l’Italia, report 2012–2016*. https://www.istat.it/it/files/2017/07/CS-Sistema-dei-conti-della-sanit%C3%A0-anni-2012-2016.pdf. (Accessed May 2021).

[27] Jonkman NH (2016). Do self management interventions in COPD patients work and which patients benefit most? An individual patient data meta-analysis. Int. J. Chron. Obstruct. Pulmon. Dis..

[28] Ministero della salute, direzione generale della programmazione sanitaria. Piano nazionale della cronicità. http://www.salute.gov.it/imgs/C_17_pubblicazioni_2584_allegato.pdf (2016). (Accessed 27 Oct 2020).

[29] Huntley AL, Johnson R, King A, Morris RW, Purdy S (2016). Does case management for patients with heart failure based in the community reduce unplanned hospital admissions? A systematic review and meta-analysis. BMJ Open.

[30] The Johns Hopkins University. *ACG System Version 11.0 Technical Reference Guide,* 11.0th edn (The Johns Hopkins University, 2014).

[31] DGR N. 498 del 16/04/13. Il sistema ACG (Adjusted Clinical Group) per la valutazione e gestione del case-mix territoriale nella Regione del Veneto. Approvazione dei risultati del primo anno (DGR 439/2012) e avvio del secondo anno e terzo anno di attività. 2013 Apr 16 (9^ legislatura).

[32] Sin BA, Akkoca O, Saryal S, Oner F, Misirligil Z (2006). Differences between asthma and COPD in the elderly. J. Investig. Allergol. Clin. Immunol..

[33] Roos LL, Walld R, Uhanova J, Bond R (2005). Physician visits, hospitalizations, and socioeconomic status: ambulatory care sensitive conditions in a Canadian setting. Health Serv. Res..

[34] Fransoo, R., Martens, P. & Burland, E. The Need to Know Team, Prior, H. & Burchill, C. *Manitoba RHA Indicators Atlas 2009* (Manitoba Centre for Health Policy, 2009).

