# Supplementary material for: How different combinations of comorbidities affect healthcare use by elderly patients with obstructive lung disease
Source: NPJ Prim Care Respir Med. 2021 May 25;31:30. doi: 10.1038/s41533-021-00242-y (PMC8149628; doi:10.1038/s41533-021-00242-y)
Supplement: Supplementary file 1 — Reporting Summary [file 41533_2021_242_MOESM1_ESM.pdf]

nature research

Corresponding author(s): DBPR NPJPCRM-01241R

Last updated by author(s): Mar 17, 2021

Reporting Summary

Nature Research wishes to improve the reproducibility of the work that we publish. This form provides structure for consistency and transparency in reporting. For further information on Nature Research policies, see our Editorial Policies and the Editorial Policy Checklist.

Statistics

For all statistical analyses, confirm that the following items are present in the figure legend, table legend, main text, or Methods section.

n/a	Confirmed	
☐	☒	The exact sample size (n) for each experimental group/condition, given as a discrete number and unit of measurement
☐	☒	A statement on whether measurements were taken from distinct samples or whether the same sample was measured repeatedly
☐	☒	The statistical test(s) used AND whether they are one- or two-sided<br>*Only common tests should be described solely by name; describe more complex techniques in the Methods section.*
☒	☐	A description of all covariates tested
☒	☐	A description of any assumptions or corrections, such as tests of normality and adjustment for multiple comparisons
☐	☒	A full description of the statistical parameters including central tendency (e.g. means) or other basic estimates (e.g. regression coefficient) AND variation (e.g. standard deviation) or associated estimates of uncertainty (e.g. confidence intervals)
☒	☐	For null hypothesis testing, the test statistic (e.g. F, t, r) with confidence intervals, effect sizes, degrees of freedom and P value noted<br>*Give P values as exact values whenever suitable.*
☒	☐	For Bayesian analysis, information on the choice of priors and Markov chain Monte Carlo settings
☒	☐	For hierarchical and complex designs, identification of the appropriate level for tests and full reporting of outcomes
☒	☐	Estimates of effect sizes (e.g. Cohen's d, Pearson's r), indicating how they were calculated

Our web collection on statistics for biologists contains articles on many of the points above.

Software and code

Policy information about availability of computer code

Data collection	Johns Hopkins ACG® System [Data collection performed by Third party]
Data analysis	R Core Team (2020). R: A language and environment for statistical computing. R Foundation for Statistical Computing, Vienna, Austria. URL https://www.R-project.org/.

For manuscripts utilizing custom algorithms or software that are central to the research but not yet described in published literature, software must be made available to editors and reviewers. We strongly encourage code deposition in a community repository (e.g. GitHub). See the Nature Research guidelines for submitting code & software for further information.

Data

Policy information about availability of data

All manuscripts must include a data availability statement. This statement should provide the following information, where applicable:

- Accession codes, unique identifiers, or web links for publicly available datasets
- A list of figures that have associated raw data
- A description of any restrictions on data availability

The data that support the findings of this study are available from AULSS 6 Euganea but restrictions apply to the availability of these data, which were used under license for the current study, and so are not publicly available. Data are however available from the corresponding author (alessandra.buja@unidp.it) upon reasonable request and with permission of AULSS 6 Euganea.

Field-specific reporting

Please select the one below that is the best fit for your research. If you are not sure, read the appropriate sections before making your selection.

☒ Life sciences ☐ Behavioural & social sciences ☐ Ecological, evolutionary & environmental sciences

For a reference copy of the document with all sections, see nature.com/documents/nr-reporting-summary-flat.pdf

Life sciences study design

All studies must disclose on these points even when the disclosure is negative.

Sample size

The sample (n. 120,181) corresponds to the entire population living within a province of Italy (and therefore being served by a Local Health Unit) and meeting these two criteria:
- age 65 or more
- suffering from an obstructive pulmonary disease.

Data exclusions

No exclusion criteria adopted.

Replication

Epidemiological study: measures taken from databases fed by administrative data. For each subject: Demographic data, chronic diseases, total healthcare costs, costs of pharmaceuticals, numbers of emergency department (ED) visits, outpatient visits and hospital admissions with main cause of admission. No replication in different settings was attempted by authors.

Randomization

We didn't control for covariates since the study's objective is mainly descriptive epidemiology / public health.

Blinding

Not relevant as this is an epidemiological study based on databases fed by administrative data.

Reporting for specific materials, systems and methods

We require information from authors about some types of materials, experimental systems and methods used in many studies. Here, indicate whether each material, system or method listed is relevant to your study. If you are not sure if a list item applies to your research, read the appropriate section before selecting a response.

Materials & experimental systems

n/a	Involved in the study	
☒	☐	Antibodies
☒	☐	Eukaryotic cell lines
☒	☐	Palaeontology and archaeology
☒	☐	Animals and other organisms
☐	☒	Human research participants
☒	☐	Clinical data
☒	☐	Dual use research of concern

Methods

n/a	Involved in the study	
☒	☐	ChIP-seq
☒	☐	Flow cytometry
☒	☐	MRI-based neuroimaging

Human research participants

Policy information about studies involving human research participants

Population characteristics

Age, sex, chronic illness.

Recruitment

The study subjects corresponds to the entire population living within a province of Italy (and therefore being served by a Local Health Unit) and meeting these two criteria:
- age 65 or more
- suffering from an obstructive pulmonary disease.

Ethics oversight

The study complied with the Declaration of Helsinki, and with resolution No. 9/2016 of the Italian Guarantor for the Protection of Personal Data, which also confirmed the allowability of processing personal data for medical, biomedical and epidemiological research, and that data concerning people's health status can be used in aggregate form in scientific studies. Permission to use unidentifiable individual data extracted from administrative databases was granted by the Azienda ULSS 6 - Euganea. To ensure confidentiality and anonymity, the Veneto Regional Authority removes all direct identifiers (e.g. NHS code numbers) and replaces them with a code number in all datasets to retain the opportunity to link data from different administrative databases.

Note that full information on the approval of the study protocol must also be provided in the manuscript.
