# Supplementary material for: How different combinations of comorbidities affect healthcare use by elderly patients with obstructive lung disease
Source: NPJ Prim Care Respir Med. 2021 May 25;31:30. doi: 10.1038/s41533-021-00242-y (PMC8149628; doi:10.1038/s41533-021-00242-y)
Supplement: Supplementary file 2 — Supplementary Information [file 41533_2021_242_MOESM2_ESM.pdf]

SUPPLEMENTARY MATERIAL

Supplementary Table 1: List of information retrieved from the ACG database

Chronic diseases, summarized with EDC (Expanded Diagnosis Cluster) codes assigned by the ACG algorithm on the basis of information collected in the previous 5 years (from 2013 to 2017)
Drug prescriptions in the year 2017, summarized with Rx-MG (Rx-Defined Morbidity Group) codes
Total healthcare costs and pharmacy costs generated in 2017
Status in life as at 31/12/2017, used for the assessment of crude mortality rates
Number of hospital admissions in 2017
Number of trips to the emergency room in 2017
Number of outpatient visits in 2017
